# Dynamic Force Modeling and Lateral Perturbation Analysis of Needle Insertion into Soft Tissues

**DOI:** 10.3390/bioengineering13030266

**Published:** 2026-02-25

**Authors:** Yao Wang, Xin Xie, Yingcai Wan, Enguang Guan

**Affiliations:** 1 Logistics Engineering College, Shanghai Maritime University, Shanghai 201306, China; wangyao@shmtu.edu.cn (Y.W.); 202330210025@stu.shmtu.cn (X.X.); 2 School of Mechanical Engineering, Shanghai Jiao Tong University, Shanghai 200240, China; wanyc_rt@sjtu.edu.cn

**Keywords:** soft tissue puncture, force modeling, biomechanical simulation, lateral perturbation, statistical analysis

## Abstract

Interface interaction mechanics analysis is of great significance for robot-assisted insertion surgery in minimally invasive surgery and therapy. Previous work indicates that the accurate modeling of soft tissue puncture forces plays a crucial role in surgical planning, robotic needle insertion, and biomechanical simulation, which can give insights useful for physicians to guide and operate assisted robots. The objective of this study is to develop a dynamic multi-component force model that integrates cutting force, stiffness resistance, and frictional interaction to characterize needle–soft tissue interaction during puncture. A dynamic force model is proposed, and a lateral periodic disturbance mechanism is introduced into the simulation framework in order to enhance the robustness and realism of the model under micro-manipulation scenarios. The model has been validated using a series of controlled puncture experiments on porcine liver and renal tissues under varying insertion angles (15°, 30°, 45°) and speeds (0.5 mm/s, 1.5 mm/s, 2.5 mm/s). Corresponding finite element simulations were also conducted using ANSYS software. The agreement between simulation and experiment has been quantitatively evaluated by comparing force–depth and force–time curves, and the statistical significance of the impact of angle and speed on puncture forces has been assessed using ANOVA and Tukey’s HSD tests. Quantitative comparison demonstrated strong consistency, with the optimal case reaching a coefficient of determination (R^2^) value of 0.96 and Root Mean Square Error (RMSE) below 0.13 N after incorporating a 0.05 mm lateral perturbation. Statistical analysis confirmed the impact of angle and speed on puncture force responses (*p* < 0.05). Furthermore, comparative analysis revealed that porcine liver exhibits more consistent biomechanical behavior than renal tissue, particularly under perturbation-enhanced simulation. This study successfully establishes a dynamic multi-component force model for soft tissue puncture, validated with high fidelity against experimental data. The incorporated lateral disturbance mechanism enhanced the model’s realism. This work can provide a reliable foundation for the future design of intelligent robot-assisted puncture systems and high-fidelity simulation-based training platforms.

## 1. Introduction

With the rapid development of medical technology and robotics, the adoption of robot-assisted or robotic surgery for minimally invasive treatment and precision medicine has become an important direction in the advancement of modern surgical procedures [1,2,3]. Needle or soft tissue puncture, as a key technology for achieving minimally invasive diagnosis and treatment, plays a fundamental role in clinical operations such as routine biopsies, local anesthesia, and proximity radiation therapy [4,5,6]. In clinical practice, whether for diagnosis or treatment, precise needle insertion is required to reach specific target areas—most of which are located within soft tissue organs. This high-precision targeting requirement is usually set at the millimeter level, and any operation outside the error margin may result in severe complications [7,8]. The main factors influencing puncture accuracy in clinical settings include human operational errors and target positioning errors [9,10,11]. These errors not only result in puncture position deviations, but may also increase the risk of surgery. Surgeons typically rely on their clinical experience and real-time visualization feedback systems to assess and adjust the puncture path or the needle trajectory [12]. Furthermore, during this process, soft tissue deformation occurs due to compression by the needle, which further increases the possibility of positioning errors [13,14].

The interaction process at the needle–soft tissue interface not only involves the nonlinear deformation and rupture behavior of soft tissues, but is also influenced by multiple factors such as insertion speed, insertion angle, needle structure/geometry, and tissue type [9,15]. A comprehensive understanding of the mechanical characteristics during the puncture, especially the variation patterns and composition of puncture forces, is crucial for optimizing puncture strategies, improving accuracy, and advancing the intelligent control of robotic puncture and surgical navigation system [16,17,18]. Although several studies have attempted to develop mechanical models of needle insertion, these models often suffer from oversimplified modeling [19], idealized tissue representations, or a lack of experimental validation [20]. Therefore, systematic experiments investigations with controlled variables are necessary to quantitatively analyze the mechanical responses during needle insertion into soft tissues. Such efforts can provide reliable data and a theoretical basis for subsequent puncture modeling and clinical applications [21,22].

The interfacial interaction mechanism between the needle and soft tissue during puncture, as well as the associated mechanics modeling, has attracted increasing attention from scholars worldwide. A brief overview of the typical literature is presented below. Okamura et al. [16] conducted systematic research on puncture force modeling. They divided the puncture process into two stages, pre-penetration and post-penetration, using experimental data, and analyzed the force acting on the needle tip at each stage. Before penetration, the needle tip is primarily subjected to rigid force from the tissue’s outer membrane. After penetration, the forces on the needle are decomposed into frictional and cutting forces [23]. Using experimental measurements and second-order polynomial fitting, Simone and Okamura [24] further established a model for the rigid force acting on the needle tip during the penetration of the outer membrane of a porcine liver sample, and employed the Karnopp approximation friction model to describe the post-penetration friction characteristics. Based on Okamura’s puncture force model, Asadian et al. [25] proposed the LuGre friction model to simulate the complex frictional characteristics of needle-tissue interactions during percutaneous intervention. This model describes the dynamic behavior of complex stick-slip friction between the needle and tissue, with the friction force distributed along the needle axis. Bora et al. [26] developed a parameterized model suitable for real-time haptic feedback in needle insertion simulations. The finite element (FE) based numerical simulations are performed to develop the parametric model, and the obtained parametric model is validated through comparison with results reported in the published literature. Recent studies have also contributed valuable insights into FE modelling of the needle interactions with soft tissues or tissue-mimicking phantoms [27,28].

Numerous scholars have also conducted extensive experimental studies on needle insertion into soft tissues. For instance, Okamura et al. [16] used bovine liver as the experimental object, employing CT imaging to measure liver tissue deformation and quantifying tissue stiffness, back membrane resistance, friction, and shear/cutting forces. Their work validated the impact of needle bevel angle and diameter on puncture forces. Due to technical limitations in laboratory conditions, such as the inability to perform real-time CT or MRI scans on biological tissues, some researchers have opted to use alternative materials with excellent optical transparency as experimental media and employ high-speed cameras to record needle and tissue deformation. DiMaio and Salcudean [29] used polyvinyl chloride (PVC) gel with embedded fiducial markers to study tissue deformation via high-speed imaging. Bao et al. [30] conducted experiments on tracheal tissue, developing a theoretical contact model for bevel-tip needles and combining FE simulations with ex vivo porcine tracheal puncture experiments to investigate insertion biomechanics. Despite these efforts, existing puncture studies remain fragmented and often domain-specific, lacking a comprehensive analysis of all relevant factors. Variations in experimental conditions and sample types result in significant discrepancies in the findings, and most works remain in the qualitative phase.

Recent studies have further advanced the characterization of tissue mechanics during insertion. For instance, Trączyński et al. [31] evaluated various force models and highlighted the significant influence of visco-elasticity at different insertion speeds. Meanwhile, sophisticated constitutive frameworks, such as the hyperelastic-based models proposed by Liu et al. [32], have been utilized to capture the nonlinear fluctuations of puncture forces. Although these high-fidelity models offer deep insights into material behavior, our proposed multi-component model achieves a practical and robust balance between physical interpretability and computational efficiency, especially under dynamic perturbation conditions.

The goal of this study is to address the current limitations by developing a comprehensive mechanical framework for needle insertion. First, the key factors influencing puncture force and the coupling between needle displacement and tissue deformation are investigated. Next, through a synergy of experimental measurements and finite element (FE) simulations, this work provides a detailed characterization of the mechanical force profiles. Furthermore, it evaluates the impact of variables including puncture angle, velocity, and tissue type on force responses, and systematically analyzes the mechanical sensitivity under lateral perturbations, thereby enhancing the model’s realism and predictive robustness. Finally, by integrating experimental data with analytical modeling, this study ensures high fidelity in puncture force predictions. These findings will aid in improving insertion precision and advancing intelligent robot-assisted systems for clinical applications.

The main contributions of this study are as follows:

• On the basis of the author’s previous work, three main stages are illustrated to describe the interaction between the puncture needle and soft tissue.
• A tri-force model for soft tissue puncture, including cutting force, friction force, and stiffness force, is proposed, covering the major mechanical mechanisms.
• A puncture experimental platform is constructed, and systematic experiments are conducted on porcine liver and kidney or renal tissues to obtain puncture response characteristics.
• Lateral periodic perturbation input is introduced into the biomechanical simulation, representing small displacement perturbations of the needle during the puncture process, enhancing the model’s adaptability and predictive capability for real scenarios.
• Comparative analyses between finite element (FE) simulation and experimental data are performed to achieve model validation and parameter optimization.

## 2. Analysis of the Soft Tissue Puncture Process

**Figure 1:**
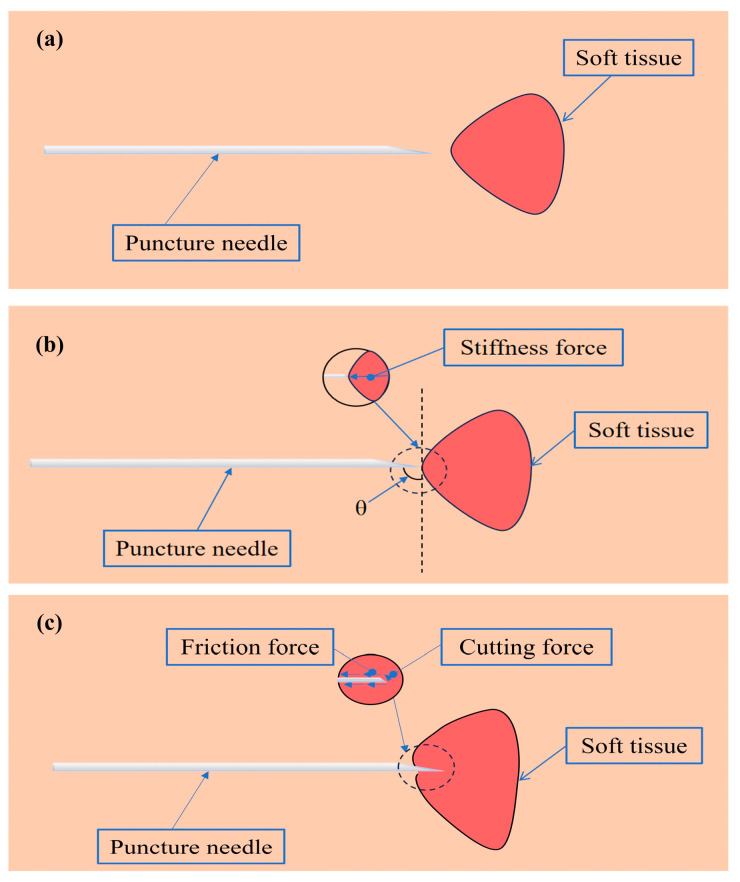
Illustration of the puncture process: (**a**) the puncture needle begins to contact with the surface of soft tissue, (**b**) the puncture needle contacts the soft tissue but does not penetrate it, and (**c**) the puncture needle penetrates the soft tissue.

Previous works of authors have developed a detailed division and analysis of the various phases of puncture process in view of the tissue deformation and puncture force [33]. So based on this, the three main stages are illustrated below to describe the interaction between the puncture needle and soft tissue, as shown in Figure 1.

1. Non-contact Stage

As shown in Figure 1a, the puncture needle has not yet made any physical contact with the target tissue surface. During this stage, the needle is approaching the target area, but no forces are applied to the tissue.

2. Contact but Not Penetrated Stage

As shown in Figure 1b, the puncture needle has just made contact with the surface of the target tissue, but has not yet penetrated it. At this moment, slight elastic deformation occurs on the tissue surface, and the needle tip experiences a reactive force from the tissue. Since the needle has not yet penetrated the outer layer, the resistance is relatively small, mainly manifesting as the tissue’s elastic response.

3. Penetration Stage

As shown in Figure 1c, the puncture needle successfully breaks through the outer layer and continues to enter into the tissue. In this stage, the needle will overcome greater resistance, originating from the inherent elasticity and structural strength of the biological tissue. As the puncture depth increases, the tissue exhibits more complex mechanical behavior.

## 3. Force Modeling of Needle Insertion into Soft Tissue

Following the classical analytical framework for needle–tissue interaction [24,29], the total puncture force is decomposed into three physically distinct components: stiffness force, frictional force, and cutting force. This tripartite division has been extensively validated in the field of robotic surgery to decouple the complex mechanical responses of soft tissues [34,35]. Specifically, (a) Cutting Force denotes the irreversible energy required for tissue fracture and crack propagation at the needle tip. (b) Stiffness Force represents the reversible elastic deformation of the tissue prior to and during penetration, primarily governed by the tissue’s hyperelastic/viscoelastic properties. (c) Frictional Force accounts for the resistance along the needle shaft due to tissue adhesion and damping effects, which varies with insertion depth and velocity. While these components are often coupled in real biological environments, this decomposition allows for a robust parameterization of the puncture process, as supported by both seminal studies [24] and recent advancements in force modeling [36,37].

The expression for the puncture force can be written as

(1) $$f_{needle}(z)\;=\;f_{cutting}(z)\;+\;f_{stiffness}(z)\;+\;f_{friction}(z)$$

where z is the puncture depth, $f_{needle}(z)$ is the puncture force, $f_{cutting}(z)\;$ is the cutting force, $f_{stiffness}(z)$ is the stiffness force, and $f_{friction}(z)\;$ is the frictional force. It should be note that $f_{cutting}(z)$, $f_{stiffness}(z)$, and $f_{friction}(z)$ are abbreviated as $f_{c}(z)$, $f_{s}(z)$, and $f_{f}(z)$, respectively.

In what follows, the modeling and analysis of each term of the formula will be explained in detail.

### 3.1. Cutting Force Modeling

During the process where the needle tip penetrates and progressively fractures the soft tissue fiber structure, the reaction force generated is called the cutting force. It originates from the fracture, tearing, and localized damage of the tissue structure, reflecting the critical energy required for tissue rupture during the puncture process.

Assuming that the needle tip has a certain sharpness during insertion and can continuously cut through the soft tissue, the cutting area is mainly concentrated at the very tip of the needle. The tissue rupture behavior can be described by the unit fracture energy, and the direction of the cutting force is collinear with the needle advancement direction but acts in the opposite direction (along the needle axis). Let the puncture angle be θ.

Let the tissue unit fracture energy be Γ, and the unit length of the cutting interface area be A(z), then the energy required for unit depth cutting is

(2) E(z)=Γ⋅A(z)

The cutting force is the derivative of energy with respect to *z*, which yields

(3) $$f_{c}(z)=\frac{dE(z)}{dz}=\Gamma \cdot \frac{dA(z)}{dz}$$

Assume that the needle tip is an ideal wedge, and the cutting area at the needle-tissue interface is proportional to the advancement depth. If the cutting width of the needle’s cross-section in the advancement direction is $w_{eff}(z)$, then

(4) $$A(z)=w_{eff}(z)\cdot z$$

So, the cutting force can be calculated as

(5) $$f_{c}(z)=\Gamma \cdot \frac{d}{dz}\left(w_{eff}(z)\cdot z\right)$$

If $w_{eff}(d)$ is a constant $w_{0}$, then

(6) $$f_{c}(z)=\Gamma \cdot w_{0}$$

This model is represented as a constant cutting force model, which is applicable to a stable puncture process. As the needle tip gradually enters the soft tissue, the initial cutting resistance increases rapidly. However, once the needle reaches the stable cutting phase, the cutting area and force do not increase indefinitely, but instead tend to stabilize.

Assume that the cutting force, $f_{c}(z)$ follows an exponential approach to saturation with puncture depth, and the model can be defined as follows:

(7) $$f_{c}(z)=f_{max}\cdot \left(\begin{array}{c} 1-e^{-\lambda z} \end{array}\right)$$

where $f_{max}$ represents the upper limit of the cutting force, and λ is the parameter controlling the rate of growth of the cutting force.

During the initial rapid ascent phase, z→0, $f_{c}(z)\approx f_{max}\cdot \lambda z$ increases linearly with the puncture depth. As it approaches saturation, $z\to \infty ,\;f_{c}(z)\to f_{max}$ reflects the tissue rupture force limit. This behavior is more consistent with the characteristics of biological tissues, where tissue rupture does not increase indefinitely, in line with the nonlinear stress–strain capacity degradation law.

### 3.2. Stiffness Force Modeling

During the puncture process of a beveled (or an angled) needle, as the needle tip enters and advances into the tissue, it encounters resistance generated by the tissue’s inherent elasticity and structural integrity. This resistance is defined as the stiffness force, which primarily arises from the elastic restoring force and nonlinear compressive behavior of the soft tissue.

The stiffness force is primarily the normal restoring force formed when the tissue resists the insertion of the needle during the interaction between the needle and tissue. Its direction is perpendicular to the tissue surface and acts on the needle tip.

Let the needle advancement depth be z, the initial stiffness of the tissue be $k_{0}$, and considering the nonlinear stiffness growth characteristics of soft tissue, it can be modeled as

(8) $$f_{s}(z)=k(z)\cdot \delta (z)$$

where k(z) represents the tissue’s equivalent stiffness coefficient, which varies with the degree of deformation; and δ(z) represents the effective strain at depth z where the tissue is compressed.

Considering soft tissue to exhibit an exponential stress–strain response, the tissue stiffness can be modeled as an exponential growth function:

(9) $$k(z)=k_{0}\cdot e^{\alpha z}$$

where α is the tissue stiffness growth coefficient.

The tissue compression, δ(z) can be estimated based on the needle tip’s slope and advancement angle. Assume that the compressed region expands along the needle tip’s normal direction. To ensure the geometric validity of the stiffness force derivation, a global coordinate system is defined where the x-y plane coincides with the undeformed horizontal surface of the tissue, and the z axis is oriented perpendicular to the surface, pointing into the tissue. The puncture angle θ is defined as the inclination angle between the needle shaft and the tissue surface. Under this convention, a perpendicular insertion corresponds to θ = 90°, which yields the maximum effective tissue compression along the insertion axis. Conversely, as θ decreases, the effective compression component varies according to the trigonometric relationship. The relationship can be expressed as

(10) δ(z)=z⋅sin(θ)

Therefore, the stiffness force can be written as

(11) $$f_{s}(z)=k_{0}\cdot e^{\alpha d}\cdot z\cdot sin(\theta )$$

### 3.3. Frictional Force Modeling

During the process of needle insertion into soft tissue, relative sliding occurs between the needle and surrounding tissue, generating frictional resistance at the interface between the surface of the needle and the inner wall of the tissue. This resistance is defined as the frictional force. The magnitude of this force dynamically changes with factors such as the needle-tissue contact length, the normal contact force, and the adhesive properties of the tissue.

The frictional force is defined as the integral of the unit length frictional stress along the contact length:

(12) $$f_{f}\left(z\right)=\int_{0}^{d}\tau \left(z\right)dz$$

where $\tau \left(z\right)$ represents the frictional stress at depth z, using the modified Coulomb model, let the frictional stress be

(13) $$\tau \left(z\right)=\mu \left(z\right)\cdot \rho_{n}(z)$$

where $\mu \left(z\right)$ represents the coefficient of friction, and $\rho_{n}(z)$ represents the normal stress at depth z on the needle wall.

If the needle is a cylinder with radius *r*, the contact area can be approximated as 2πrdz, and the total frictional force can be written as

(14) $$f_{f}\left(z\right)=\int_{0}^{d}\;\mu \left(z\right){\cdot \rho }_{n}\left(z\right)\cdot 2\pi rdz$$

If we further assume that the normal stress, $F_{s}(z)$ is derived from the projection of the stiffness force, i.e., $p_{n}(z)\approx \frac{f_{s}(z)}{2\pi r\cdot dz}$, and $\mu (z)=\mu_{0}$ is a constant. Therefore, the frictional force is simplified as

(15) $$f_{f}(z)=\mu_{0}\int_{0}^{d}\;F_{s}(z)dz$$

By substituting the previously derived stiffness force model, i.e., Equation (11), the following expression can be obtained:

(16) $$f_{f}(z)=\mu_{0}\cdot k_{0}\cdot sin(\theta )\cdot \int_{0}^{d}\;ze^{\alpha z}dz$$

Then, the integral has an analytical solution, which gives

(17) $$f_{f}(z)=\mu_{0}\cdot k_{0}\cdot sin(\theta )\cdot \left(\frac{e^{\alpha d}(\alpha d-1)+1}{\alpha^{2}}\right)$$

In summary, Equations (7), (11), and (17) will be substituted into Equations (1) to solve and calculate the puncture force.

### 3.4. Parameter Identification

The characteristic parameters of the proposed multi-component model, including the cutting force parameters and stiffness parameter, were determined through non-linear least-squares regression based on the experimental data collected at 0.5 mm/s. The optimization was performed using the Levenberg–Marquardt algorithm in MATLAB R2022a with the Curve Fitting Toolbox. To ensure the reliability of the model, the identification process aimed to minimize the RMSE between the analytical prediction and the experimental curves. The 0.5 mm/s dataset was selected for parameter identification to minimize the influence of dynamic effects and high-speed viscoelastic fluctuations, providing a stable baseline for the stiffness and cutting force components. The identified parameter values are summarized in Table 1.

**Table 1:** Identified parameters for the multi-component force model across different tissue types.

Tissue Type	$f_{max}$ (N)	λ (mm^−1^)	$k_{0}$ (N/mm)	α (mm^−1^)
Porcine Liver	1.68	0.12	0.05	0.21
Porcine Renal Tissue	1.12	0.15	0.08	0.25

## 4. Needle–Soft Tissue Puncture Experiments

To validate the analyses presented in Section 3 and to determine the appropriate effective values for puncture or insertion speed and angle, this study conducted a series of needle-soft tissue puncture experiments. The objective was to improve puncture accuracy, ensure puncture performance, and achieve optimal therapeutic outcomes.

### 4.1. Experimental Equipment

**Figure 2:**
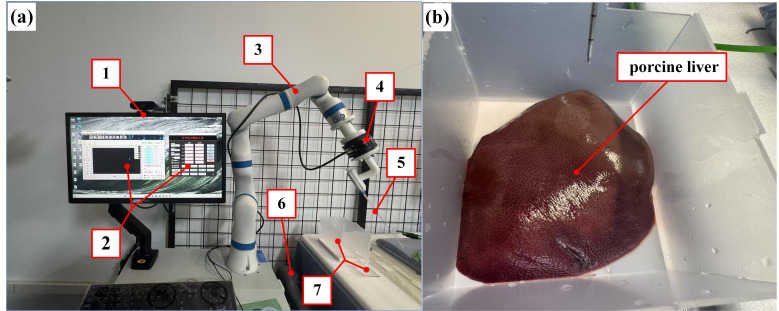
Experimental setup and biological tissue used for puncture events: (**a**) puncture experiment platform, and (**b**) porcine liver specimen. The numbers denote the following: 1—host computer, 2—data acquisition system, 3–7—DOFs robotic arm, 4—force sensor, 5—puncture needle, 6—teach pendant, and 7—container used for holding experimental specimen.

Figure 2a illustrates the experimental setup, which features a custom-developed puncture platform. The entire puncture platform mainly consists of a 7-DOF (or seven-axis) robotic arm, a host computer, a teach pendant, a force sensor, and a needle module with clamp. By adjusting the robotic arm parameters through the teach pendant, the platform enables precise control over both the puncture speed and angle during needle insertion into soft tissue.

A 7-DOF robotic arm from Tianlian Robotics Co., Ltd. (Mainyang, China) was employed in this experiment. To maintain a consistent puncture angle without inducing motion in other joints, the arm was operated using a tool coordinate system. In this configuration, the Z-axis was defined as the effective direction of the tool mounted at the robot’s wrist, with the coordinate origin located at the tool tip. The system distinguishes between basic axes (X, Y, Z) for translational motion and orientation axes (A, B, C) for rotational motion. Specifically, TA, TB, and TC denote rotations about the tool’s TX, TY, and TZ axes, respectively. This tool-centered coordinate framework ensures that motion commands are executed relative to the tool’s orientation, remaining unaffected by changes in the robot’s base position or overall configuration.

Moreover, the six-dimensional force sensor used in this experimental platform is the Y82 series six-component force sensor from DST Sensing System Engineering Co., Ltd. (Shenzhen, China), which is capable of measuring 6 degrees of freedom (i.e., 6-DOFs), including three linear forces (Fx, Fy, Fz) and three torque components (Tx, Ty, Tz). The sensor is accompanied by two software tools: the D.R304 debugging tool and the D.R304 testing and recording system. The D.R304 debugging tool uses RS-485 communication for parameter configuration and IP address modification, while the D.R304 testing and recording system uses Ethernet communication for high-speed data monitoring. It also supports features such as curve plotting and data storage, meeting the need for high-precision, multidimensional force data acquisition during experiments.

Although a 6-DOF force sensor was employed to capture the full wrench of the interaction, the measured lateral forces (Fx, Fy) were consistently an order of magnitude smaller than the axial force (Fz). Across all tested puncture angles and speeds, the peak magnitudes of Fx and Fy were observed to be less than 10% of the maximum Fz. These observations align with previous experimental studies [33,37], which reported a similar magnitude disparity (approximately 1:15 ratio) between lateral and axial forces in soft organ puncture. Consequently, focusing on the axial force components enables a robust and computationally efficient model that captures the essential mechanics required for real-time surgical assistance, without compromising the overall accuracy of the puncture force prediction in the primary direction of motion.

### 4.2. Experimental Design

This study aims to systematically investigate the effects of puncture speed and puncture angle on the mechanical response generated during the puncture process of soft tissues. Considering that, in practical clinical puncture and interventional operations, tissue type, operation angle, and advancement speed all significantly impact the magnitude and variation trends of the puncture force, this experiment uses different types of soft tissues as subjects, setting multiple speed and angle combinations. Force–displacement and force–time response tests are conducted to reveal the regulatory effects of these factors on the composition, peak value, and variation trends of the puncture force. The specific puncture speeds and angles are shown in Table 2.

In this study, a fixed-factor experimental design was employed to investigate the effects of two factor groups—puncture speed and puncture angle—on puncture deviation, and to identify the optimal value range of the relevant parameters. For the analysis of puncture speed, experiments were conducted under a fixed puncture angle of 90°. The robotic arm was adjusted such that the needle was aligned perpendicularly to the soft tissue surface, ensuring that the needle axis passed through the predefined target point at the start of each insertion. Conversely, to evaluate the effect of puncture angle, the puncture speed was held constant at 1.5 mm/s. To minimize measurement error in the evaluation of puncture deviation, each experimental condition was repeated 5 times. The deviation values obtained from these repeated trials were averaged to represent the final result for each group.

The selection of puncture velocities and insertion angles was based on typical clinical protocols for percutaneous interventions. Taking percutaneous lung puncture surgery as an example, in clinical practice, the procedure was usually performed via the anterior axillary line, midaxillary line, posterior axillary line, or angulus inferior scapulae line, and the needle was needed to pass through human skin, muscle, parietal pleura, pleura cavity, visceral pleura, etc., and the insertion depth of the entire puncture path was about 20 to 80 mm considering the individual difference of the patient. Meanwhile, clinical practice showed that the actual time for a doctor to perform the insertion action is about 2–3 min, so since the continuity of the robot action, the minimum value of puncture speed is set to 0.5 mm/s, and the maximum value is generally not more than 2.5 mm/s. The puncture angle was determined based on empirical values derived from surgical situations documented in existing medical records. Specifically, the angles of 15°, 30° and 45° were chosen as they represent the typical clinical range of percutaneous needle insertion, and this selection was also informed by considerations of spatial symmetry and surgical path planning, enabling a systematic analysis of the angle dependent mechanical response. Overall, the approximate value ranges for each factor were derived from the related published literature, or the clinical experience of doctors, and the results of authors’ previous works. Low velocities were chosen to capture quasi-static behavior and minimize inertial effects, while the angular range represents common entry trajectories used in liver and kidney biopsies to avoid critical vascular structures.

### 4.3. Experimental Procedure and Result Analysis

**Figure 3:**
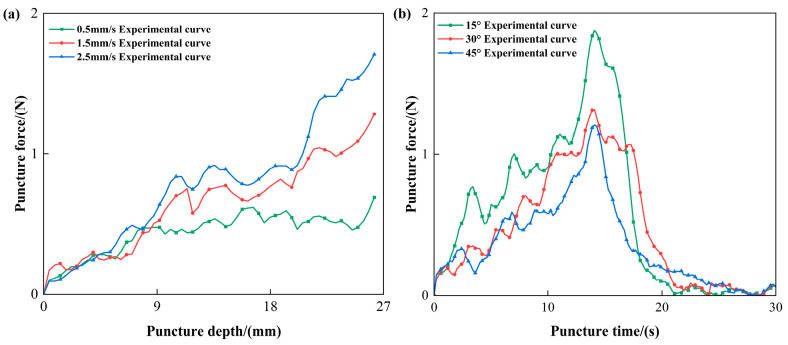
Effect of puncture speed and angle on force response during porcine liver puncture: (**a**) puncture force–depth curves under different puncture speeds, and (**b**) puncture force–time curves under different puncture angles.

Fresh porcine liver tissue was selected as the puncture target to simulate the mechanical response of needle–tissue interactions, as illustrated in Figure 2b. The specimens were sourced from a local slaughterhouse and utilized within 4 h post-slaughter to ensure structural integrity and optimal bio-mechanical properties. To maintain tissue’s viability, the specimens were initially stored at 4 °C and then gradually equilibrated to the laboratory temperature of 22 °C ± 2 °C prior to testing, thereby minimizing temperature-induced variations in the subsequent puncture force. The porcine liver specimen dimensions were approximately 10 cm × 10 cm × 5 cm. This configuration effectively suppressed lateral slippage and macro-scale deformation during needle insertion, ensuring that the experimental boundary conditions remained consistent with the ‘fixed ends’ assumption employed in the Finite Element Analysis (FEA). A stainless steel hollow bevel needle, with a diameter of 1.2 mm (i.e., 18 G), was employed for all trials. Bevel-tipped needles were chosen due to their sensitivity to tissue anisotropy and their prevalence in clinical practice. As shown in Figure 3, preliminary experimental results indicate that the puncture force correlates positively with insertion velocity, while demonstrating a significant inverse relationship with the insertion angle.

Figure 3 illustrates the variation in puncture force under different puncture speeds and angles. In Figure 3a, the puncture force generally increases in a stepwise manner with depth, and higher puncture speeds require greater force at the same depth, indicating that faster speeds may induce stronger tissue resistance. Figure 3b illustrates that under all tested puncture angles, the insertion force increases and reaches a peak within the 10–20 s interval, followed by a rapid decrease. This trend reflects the complete insertion followed by the smooth retraction of the needle from the tissue. The force profile indicates that puncture angle significantly influences the peak insertion force, with the highest force observed at a 15° puncture angle, suggesting greater resistance at this orientation. As highlighted in Figure 3b, the peak force at a 15° puncture angle significantly exceeds that at a 45° puncture angle. This phenomenon is attributed to the path-length effect within the high-stiffness superficial layers. At shallow angles, the needle traverses a substantially longer distance through the fibrous outer capsule and the dense cortical layer before reaching the more compliant medullary tissue. The accumulation of cutting resistance and lateral squeezing forces along this extended path results in elevated force peaks compared to the more direct, shortened trajectory at 45°.

To gain a deeper understanding of the mechanical mechanisms involved in the interaction between the puncture needle and soft tissue, this study employs an integrated approach combining simulation modeling (i.e., FE simulation) with in vitro experiments. To this end, a FE-based soft tissue puncture model is developed to simulate the nonlinear deformation and fracture processes as the needle penetrates at different speeds and angles. Meanwhile, in vitro puncture tests are performed on various tissue samples to obtain force–displacement and force–time curves, enabling detailed analysis of the force components and their variation patterns. The experimental and simulation results are then compared to evaluate the consistency of the mechanical response characteristics, thereby verifying the model’s reliability and improving its capability to predict the behavior of real biological tissues.

### 4.4. Analysis of Simulation Results

The simulation was performed using ANSYS 2022 R1 on a workstation equipped with an Intel Core i9-13900K CPU @ 3.0 GHz and 32 GB of RAM. To balance accuracy and efficiency, each simulation case required an average computation time of approximately 3 h. Due to the heterogeneity, anisotropy, viscoelasticity, and contact nonlinearity of the soft tissue [33,36,37], it is difficult to calculate the needle force. So, the soft tissue material is simplified in this study, assuming that the soft tissue under different material properties is an isotropic hyperelastic material.

To accurately simulate the large deformations during the puncture process, the soft tissues were modeled as hyperelastic materials in ANSYS using the Mooney-Rivlin constitutive model. This model is well-regarded for its capacity to characterize the nonlinear mechanics of biological deformable bodies. The material parameters were derived from established literature [38]. Specifically, the equivalent Young’s modulus was set to 2 MPa for porcine liver, with a density of 1100 kg/m^3^. A Poisson’s ratio of 0.45 was adopted to reflect the near-incompressibility of biological soft tissues.

**Figure 4:**
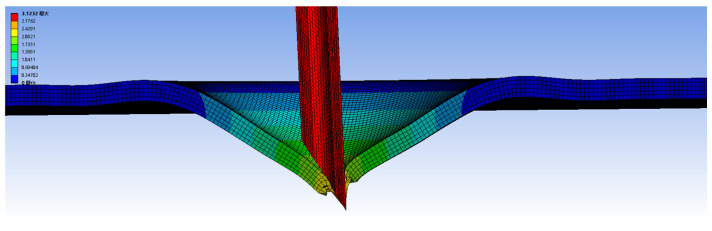
Finite element simulation of needle insertion into soft tissue. Note that the non-English term ‘最大’ in the figure indicates ‘Maximum’, ‘最小’ in the figure indicates ‘Minimum’.

Regarding the simulation setup, the tissue model was constrained by fixing both ends to define the boundary conditions. To ensure computational accuracy and accommodate large strains, the soft tissue and needle were discretized using hexagonal adaptive mesh elements, with further mesh refinement applied to the contact analysis region. The interaction between the needle and tissue was modeled with a friction coefficient of 0.2 [39], and tissue failure was defined based on a unit damage failure mode. The resulting deformation distribution is illustrated in Figure 4.

To enhance the realism and applicability of the needling force model, this study introduces a lateral disturbance mechanism, simulating the impact of micro-vibrations from the robotic arm and human operational errors on the needling process. The small vibrations of the robotic arm are typically caused by control precision, external disturbances, and robot execution accuracy. These vibrations may lead to slight displacement of the needle during insertion, affecting the contact force between the needle and soft tissue, as well as the insertion depth. Although these vibrations are usually small, they can significantly impact the precision of the needling process, especially in micro-manipulation and high-precision needling tasks.

Additionally, existing research indicates that the movement of the needle within soft tissue is affected by small vibrations [16]. Compared to these studies, we find that incorporating the lateral disturbance model effectively enhances the realism of the simulation results, especially when simulating microscopic uncertainties during the surgical process.

Specifically, in the ANSYS explicit dynamics module, a Remote Displacement boundary condition is applied at the rear end of the needle, with linear progression displacement applied along the Z-axis (puncture direction), while a periodic disturbance function is applied along the X-axis (lateral direction).

(18) $$u_{x}(t)=A\cdot sin(2\pi ft)$$

where A=0.05 mm is the disturbance amplitude, f=1000 Hz is the disturbance frequency, and *t* is the simulation time.

In Equation (18), the disturbance frequency f is set to 1000 Hz to align with the sampling bandwidth of the force sensor, serving as a rigorous stress test for numerical stability.

It is worth noting that, despite this high-frequency lateral input, the axial force response remains stable. This is attributed to the orthogonal nature of the disturbance relative to the insertion axis. Since the perturbation amplitude is significantly smaller than the puncture depth and is applied laterally, its influence on the macroscopic axial force is minimal compared to the dominant cutting and stiffness forces. Consequently, this validates that the proposed model is structurally robust: it effectively captures the primary mechanical response of tissue puncture without being destabilized by high-frequency, low-amplitude lateral noise.

To implement the lateral perturbation defined in the ANSYS environment, a tabular data input method was employed. Since the explicit dynamics module requires discretized inputs for complex time-varying loads, the sinusoidal disturbance function was pre-processed into a series of discrete time-displacement data points. These values were then imported into the Remote Displacement boundary condition table (X-component) to drive the lateral motion of the needle.

**Figure 5:**
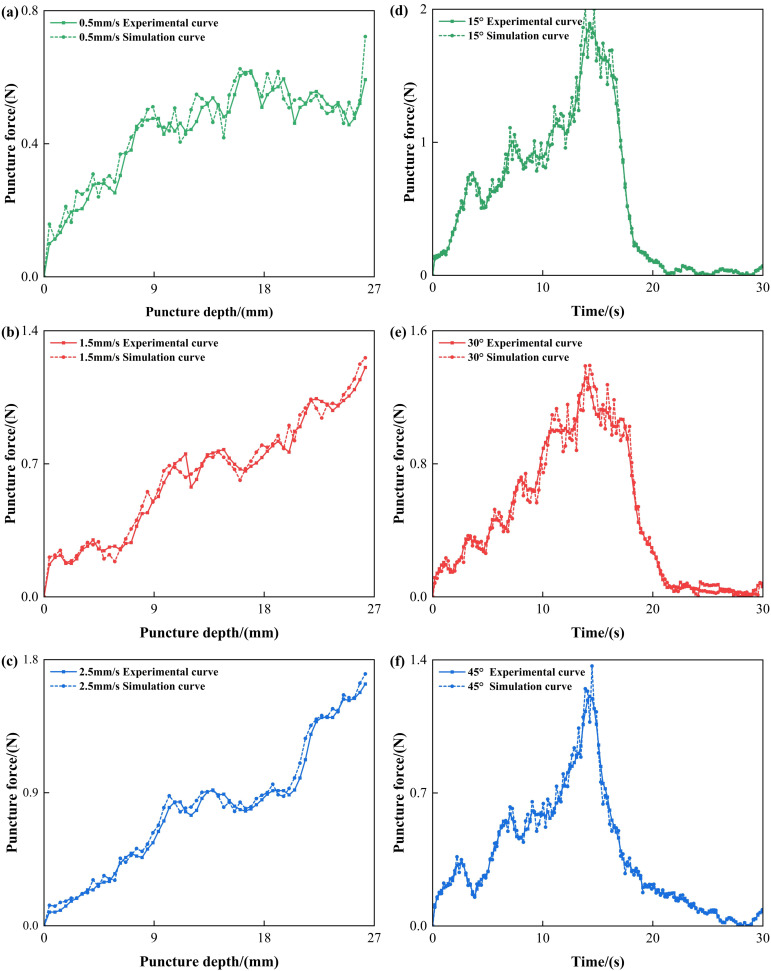
Comparison of experimental and simulation results of porcine liver puncture force at different speeds and angles: (**a**–**c**) puncture force–depth curves for experimental and simulation data under different puncture speeds, and (**d**–**f**) puncture force–time curves for experimental and simulation data under different puncture angles.

To verify the accuracy of the developed puncture simulation model, this study compares the simulation results of puncture speed and puncture angle curves with experimental data, as shown Figure 5a–f. The figure shows the force response trends in both the time and depth domains, directly comparing the simulated and experimental curves under the same puncture conditions (i.e., same speed and angle).

The results demonstrate strong agreement between the simulation and experimental curves across the key phases of the puncture process, specifically:

• Initial Contact Stage:

The simulation accurately captures the slow increase in stiffness force as the puncture needle comes into contact with the soft tissue, mirroring the experimental trend.

• Rupture Stage:

The simulation successfully reproduces the sharp force rise observed in the experimental data, indicating that the implemented erosion or rupture failure mechanism is effective and appropriately modeled.

• Withdrawal and stabilization Stage:

The simulation curve exhibits a force recovery and plateau similar to that of the experimental results, reflecting the sustained frictional interaction between the needle and the tissue during the withdrawal or stabilization stage.

**Figure 6:**
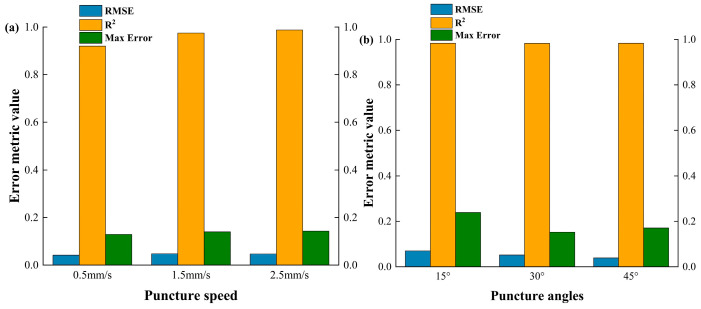
Comparison of simulation errors in porcine liver puncture under different (**a**) puncture speeds and (**b**) puncture angles: RMSE, R^2^, and Max Error. Note that the left Y-axis represents the values for RMSE and Max Error, while the right Y-axis corresponds to the R^2^ values.

To further quantitatively evaluate the differences between the simulation and experimental results, the root mean square error (RMSE) and the coefficient of determination (R^2^) between the two were also calculated, as shown in Figure 6a,b.

The results demonstrate that, under varying puncture speeds and angles, the porcine liver puncture simulation model exhibits consistently high predictive accuracy. The R^2^ remains close to 1 across all conditions, indicating that the simulations effectively capture the overall trends of the experimental force–displacement curves. The RMSE values are both low and stable, suggesting that the average prediction error is minimal. The maximum error also remains relatively small, occurring primarily during localized events such as force peaks, with a slight decrease observed at larger puncture angles. These findings confirm the robustness and stability of the model across diverse operating conditions, with only limited deviations present during transient tissue fracture phases.

### 4.5. Analysis of Results from Different Components of Soft Tissue

**Figure 7:**
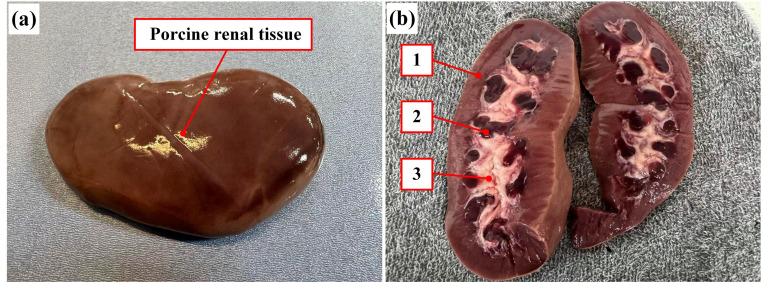
Porcine renal tissue anatomy and structure components: (**a**) whole porcine renal tissue specimen, and (**b**) the internal structure components of renal. The numbers denote different components as follows: 1—renal cortex, 2—renal medulla, and 3—renal calyx.

Furthermore, to further investigate the mechanical response characteristics of soft tissues during the puncture process, comparative experiments were conducted on porcine renal tissue following the completion of the porcine liver tests. The porcine renal tissue was specifically selected due to its well-defined layered anatomical structure, which facilitates clearer differentiation of force transitions across distinct puncture phases. And its structural and mechanical resemblance to the human renal, including similarities in tissue density, capsular composition, and internal organization, makes it a representative model in urological surgical simulations and puncture trajectory planning. By measuring puncture forces under varying angles and speeds, the influence of tissue specific properties on puncture behavior can be further elucidated. The porcine renal tissue and its internal anatomy are illustrated in Figure 7a,b.

To gain a deeper understanding of the impact of different tissue structures on the reaction force during the puncture process, this study conducts a segmented analysis of the puncture force–depth curve. Based on the anatomical structure changes along the insertion path and their corresponding curve characteristics, the entire process is divided into three ranges: Component 1 (Renal Cortex), Component 2 (Renal medulla), and Component 3 (Renal Calyx).

**Figure 8:**
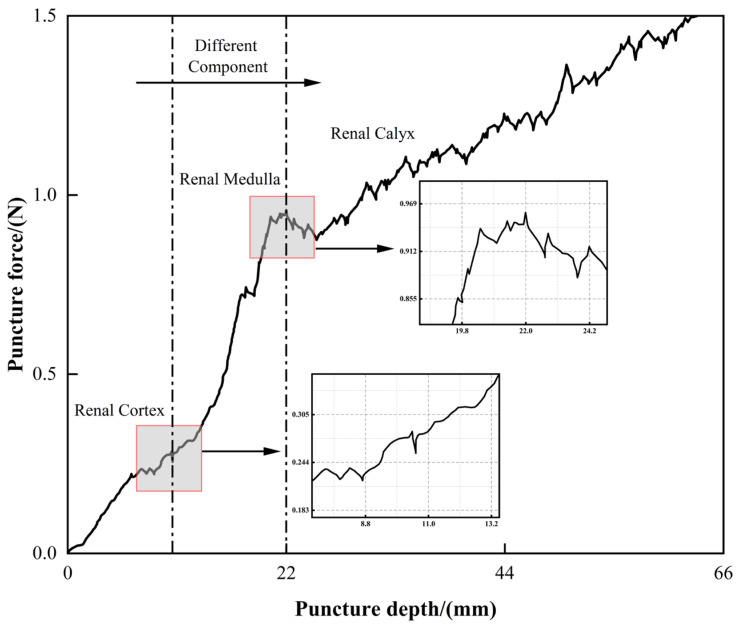
Puncture force–depth curve for porcine renal tissue with segmentation into different components.

Based on the puncture force–depth curve for porcine renal tissue, the puncture process can be divided into three stages, each exhibiting different mechanical characteristics as shown Figure 8.

In component 1, the puncture force shows a slow, linear increase. The renal cortex is dense, rich in capillaries, and has a uniform structure. During the puncture, the force is primarily influenced by soft tissue rupture resistance and some tissue adhesion. As a result, the puncture force increases steadily with almost no significant fluctuations.

In component 2, the puncture force increases sharply, showing a steep rise. This stage corresponds to the renal medulla, which is denser and more rigid. The needle must overcome higher fracture energy, resulting in a significant increase in cutting force and the occurrence of local force peaks. The non-uniformity of the tissue structure and local fractures cause the puncture force to fluctuate more in this stage.

In component 3, the puncture force increases slowly. The renal calyx consists of a cavity or is filled with low-density fluid, with an inner lining of smooth mucosal tissue. As the needle enters this region, tissue resistance decreases significantly, weakening the cutting force while friction becomes the primary reaction force. The force variation becomes smaller, and the puncture process stabilizes.

In short, the Renal Cortex range is dominated by stable cutting with lower puncture force, the Renal medulla range is where resistance sharply increases with more force fluctuations, and the Renal Calyx range is dominated by friction with minimal force changes. The different tissue structures (i.e., different components of soft tissue) significantly affect the puncture force, providing a theoretical basis for the optimization of puncture models and path planning.

While real-time imaging was not employed during insertion, the boundaries between the cortex, medulla, and calyx were identified by correlating the force–gradient fluctuations with established anatomical thickness data for porcine kidneys. The distinct change in the slope of the force–depth curve serves as a mechanical signature for the transition between tissue layers of varying densities.

Similarly, an isotropic hyperelastic material model is used to account for the porcine renal tissue, with modeling based on the tissue structural characteristics of the renal tissue. Different components of the renal tissue exhibit distinct mechanical behaviors, so different Young’s modulus and Poisson’s ratios are assigned to these regions to accurately describe their mechanical properties [38]. Specifically, the Young’s modulus of the renal cortex is set at 10 MPa, with a Poisson’s ratio of 0.4; the Young’s modulus of the renal medulla is 5 MPa, with a Poisson’s ratio of 0.35; and the Young’s modulus of the renal calyx is lower, is 2 MPa, with a Poisson’s ratio of 0.35.

**Figure 9:**
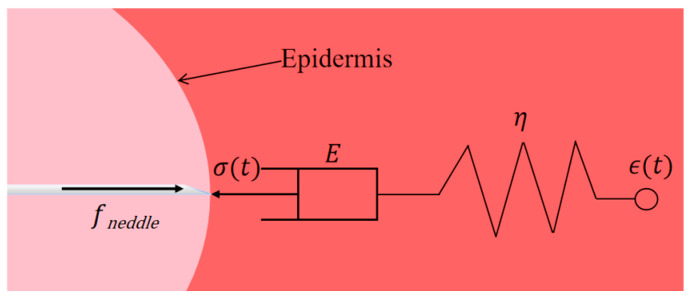
Maxwell’s model used in the simulation for viscoelastic behavior.

In addition, in order to characterize the viscoelastic behavior of different components of porcine renal tissues, the Maxwell model is introduced and employed to simulate the stress relaxation and creep behavior of the porcine renal tissue during the needle insertion process. This model combines elastic and viscous components, describing the stress–strain relationship of the material by connecting a spring and a damper in series. In this model, the stress not only depends on the elastic deformation of the tissue (represented by the spring), but also takes into account the viscous resistance of the tissue during deformation (represented by the damper). The specific schematic diagram is shown in Figure 9.

The basic form of the Maxwell model can be expressed as

(19) $$\sigma (t)=E\cdot \epsilon (t)+\eta \cdot \frac{d\epsilon (t)}{dt}$$

where σ(t) is the stress at time t, E is the elastic modulus (i.e., Young’s modulus), ϵ(t) is the strain at time t, η is the viscosity coefficient (indicating the resistance of the damper), $\frac{d\epsilon (t)}{dt}$ is the strain rate.

In this model, the material’s stress is not only related to its elastic deformation, but also influenced by its viscous resistance. In this way, the Maxwell model accurately describes the material’s stress response across different time scales, especially during the needle insertion process, capturing the nonlinear and strain-hardening characteristics of viscoelastic materials.

The selection of constitutive models is tailored to the specific mechanical characteristics of the target organs. The Mooney–Rivlin model is employed for the liver to primarily characterize its nonlinear hyperelastic deformation during large-scale compression [40]. Conversely, for the kidney or renal tissue, the Maxwell model is introduced to specifically account for the viscoelastic stress relaxation and time-dependent behavior observed during multi-layer penetration, which is based on both published literature [41,42] and theoretical considerations. Firstly, drawing on relevant findings from the recent published literature, Mishra A et al. [41] investigated the viscoelastic properties of porcine kidney in the upper, middle and lower poles using oscillatory shear tests, and found that the Maxwell model is an effective choice for capturing the frequency dependence of storage modulus and loss modulus. Next, from a physical perspective, kidney or renal tissue exhibits a rapid elastic response upon needle insertion, followed by gradual stress relaxation due to different components or interstitial fluid redistribution—a behavior naturally described by the Maxwell model’s spring-dashpot series configuration. Finally, the simplicity of selected model ensures computational efficiency in FE simulations without sacrificing essential physical fidelity. Therefore, by combining these approaches mentioned above, the established model in this study can capture both the instantaneous elastic resistance and the subsequent force decay, ensuring a comprehensive representation of tissue-needle interaction.

**Figure 10:**
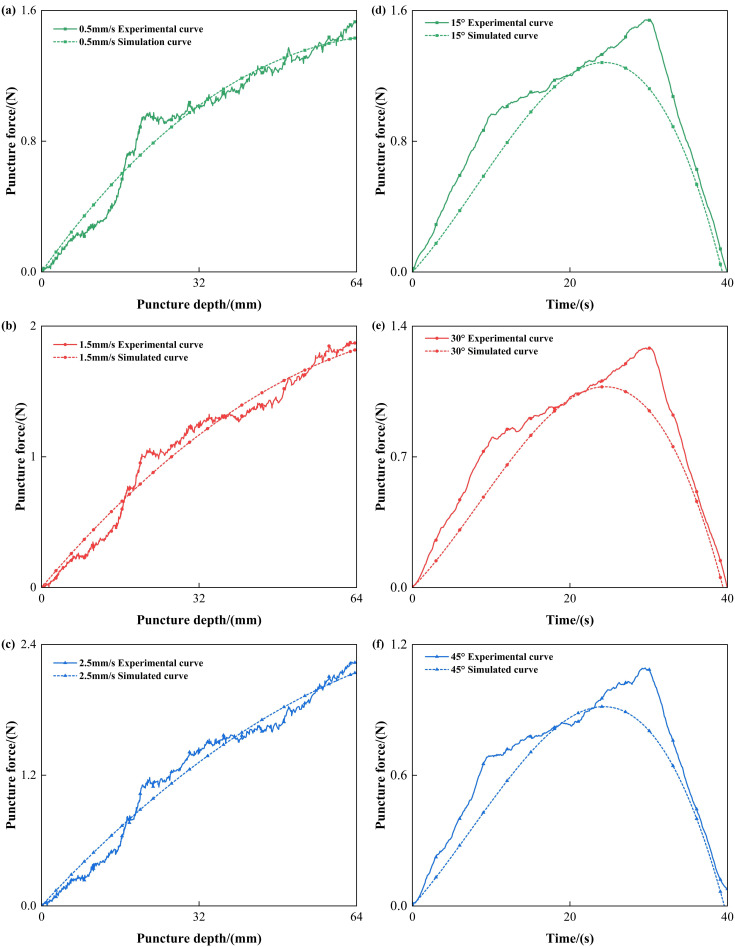
Comparison of experimental and simulation results of porcine renal tissue puncture force at different speeds and angles: (**a**–**c**) puncture force–depth curves for experimental and simulation data under different puncture speeds, and (**d**–**f**) puncture force–time curves for experimental and simulation data under different puncture angles.

To verify the reliability of the simulation model in reproducing the puncture behavior of porcine renal tissue, a comparison between experimental and simulated results was performed, as shown in Figure 10a–f.

As shown in Figure 10, the simulation curves generally exhibit similar trends to the experimental curves across different puncture speeds and puncture angles. In all cases, the puncture force increases with depth or time until reaching a peak, followed by a decline, indicating that the model effectively captures the overall mechanical response during the penetration process. However, noticeable discrepancies are observed. In most cases, the simulated peak forces are slightly lower than the experimental values, particularly at larger puncture angles, and the post-peak force drop in the simulation is faster and smoother. In contrast, the experimental curves exhibit more gradual and fluctuating declines, likely due to the complex fracture modes, frictional effects, and structural heterogeneity of real biological tissues.

The influence of puncture speed is consistent in both simulation and experiment, with higher speeds resulting in larger peak forces and slightly increased fluctuations. The simulation captures this trend well, although the peak forces at higher speeds are underestimated. Regarding the puncture angles, both simulation and experimental results show that larger angles led to lower peak forces and shorter times to reach the peak, which can be attributed to changes in the effective contact area of the needle tip. The discrepancies between simulation and experiment are more pronounced at larger angles, suggesting that the current model may require further refinement to account for anisotropy, viscoelasticity, and speed-dependent friction effects.

**Figure 11:**
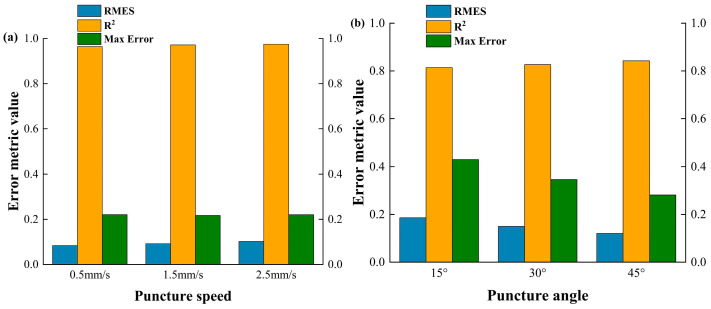
Comparison of simulation errors in porcine renal tissue puncture under different: (**a**) puncture speeds and (**b**) puncture angles: RMSE, R^2^, and Max Error. Note that the left Y-axis represents the values for RMSE and Max Erro, while the right Y-axis corresponds to the R^2^ values.

Similarly, to further evaluate the accuracy of the simulation, a comparison of simulation errors under different puncture conditions (puncture speed and angle) is presented in the figure below. Figure 11a,b compares the RMSE, R^2^, and maximum error values for different puncture angles.

As shown in Figure 11, it reveals that, for porcine renal tissue puncture, the simulation model achieves strong agreement with experimental results across all tested puncture speeds and angles. The R^2^ values remain high, consistently above 0.9, confirming the model’s reliability in reproducing experimental force profiles. Compared with the porcine liver results, the RMSE values are slightly higher, suggesting a marginally larger average deviation, which may be attributed to the more complex internal structure and anisotropy of renal tissue. The maximum error values are also relatively higher than those observed in the liver case, particularly at lower puncture speeds and smaller angles, reflecting greater local discrepancies during rapid force transitions. Overall, the model demonstrates good predictive capability for renal tissue, though with slightly reduced precision in capturing localized mechanical responses compared with liver puncture simulations.

To further investigate the effects of varying puncture speeds and angles on the outcomes of porcine liver and porcine renal tissues puncture experiments, we also conducted a statistical analysis comprising a one-way Analysis of Variance (ANOVA), followed by Tukey’s Honestly Significant Difference (HSD) post hoc test. The objective was to assess whether the observed differences between experimental groups were statistically significant. A total of 12 pairwise comparisons were performed. For instance, comparison a_1_ evaluates the difference between porcine liver puncture speeds of 0.5 mm/s and 1.5 mm/s; a_2_ compares 0.5 mm/s with 2.5 mm/s; a_4_ examines the difference between puncture angles of 15° and 30°, and so forth. Each comparison yields a mean difference (meandiff), an adjusted *p*-value (to control the family-wise error rate), and a 95% confidence interval, specified by its lower and upper bounds.

The meandiff represents the numerical difference between the average outcomes of the two groups under comparison. Specifically, it is calculated as the mean of the first group minus the mean of the second group. A positive value indicates that the first group has a higher mean outcome than the second, whereas a negative value suggests the opposite. Statistical significance is determined based on the adjusted *p*-value and whether the confidence interval includes zero. If the adjusted *p*-value is below the conventional threshold (typically *p* < 0.05) and the confidence interval does not span zero, the null hypothesis of no difference is rejected, indicating a significant difference between the groups. The results are summarized in Table 3 including the mean differences, adjusted *p*-values, confidence intervals, and the statistical decision (i.e., whether the difference is significant).

According to the results of the ANOVA and Tukey HSD test, it can be concluded that different puncture speeds and angles have significant effects on the experimental results of porcine liver and porcine renal tissues, so choosing the right speed and angle is crucial to ensure the accuracy and repeatability of the experimental results. These significant differences provide an important basis for further optimization of experimental settings and research.

## 5. Discussion

In this study, porcine liver and porcine kidney or renal tissues were modeled as isotropic hyperelastic material to simplify the puncture mechanics. However, actual biological tissues often exhibit significant anisotropy and heterogeneity, which were not fully considered in the current model. For example, soft tissues like the liver and renal tissues may show different mechanical behaviors in different directions, which can affect the mechanical response during needle insertion. Therefore, future studies should consider introducing anisotropic material models to more accurately describe the mechanical properties of soft tissues. To improve the accuracy of the model, it is recommended to use orthotropic or transversely isotropic material models and parametrize the tissue’s anisotropic properties with experimental data. This approach would enable the model to better simulate the mechanical behavior during the actual puncture process, especially when dealing with complex tissue structures, thereby enhancing the accuracy and applicability of the simulation.

As observed in Figure 10d–f, the simulated force profile exhibits a more rapid decline during the retraction phase compared to the experimental data. This discrepancy suggests that the modified Coulomb friction model used in the simulation may not fully account for the viscous adhesion and asymmetric tissue recoil that occur during needle withdrawal. Future iterations of the model will incorporate rate-dependent friction laws to better characterize this hysteretic behavior.

The puncture process of porcine liver and porcine renal tissues at different puncture speeds and puncture angles was studied using both experimental and simulation methods. The results indicate that both puncture speed and puncture angle significantly affect the puncture force. Specifically, as the puncture speed increases, the force required for puncture also increases, and the force–depth curve becomes steeper. Conversely, as the puncture angle increases, the force required for puncture decreases, and the force–time curve becomes smoother. These findings are consistent with the understanding that puncture speed and puncture angle directly influence the force needed to overcome tissue resistance.

Moreover, the influence of different tissue structures on puncture force was evident from the experiments. The porcine renal tissue, which has distinct anatomical regions such as the renal cortex, renal medulla, and renal calyx, displayed varying mechanical responses to different ranges of punctures. The renal cortex, being dense and rich in capillaries, showed relatively stable cutting forces, while the renal medulla, with its higher tissue rigidity and non-uniform structure, caused a sharp increase in force, followed by more fluctuation. The renal calyx, with its smoother mucosal tissue and lower-density fluid, showed significantly reduced puncture forces.

While the simulation results generally agreed with the experimental data, certain discrepancies were observed, particularly at greater puncture depths. The simulation model, which was based on simplified material properties and idealized assumptions, did not fully account for the complex mechanical characteristics of porcine renal tissue. In particular, the nonlinear elasticity, viscoelastic behavior, and the potential deformation of the tissue during the cutting process were not sufficiently captured. These factors likely contributed to the larger errors in the simulation results as the puncture depth increased.

Additionally, the introduction of lateral disturbances (e.g., from mechanical arm micro-vibrations) could have influenced the puncture process. While we modeled these disturbances in the simulation, further improvements in the disturbance modeling could help refine the simulation’s ability to predict real-world puncture behavior, especially in dynamic environments. To further investigate the impact of lateral disturbance amplitude on simulation accuracy and assess the effects of different angles and speeds on the model’s performance, we utilized a unified error metric for quantitative comparison. By employing standardization and weighted aggregation methods, we harmonized the error metrics across various angles and speeds, ensuring that all evaluations had the same dimensionality.

**Figure 12:**
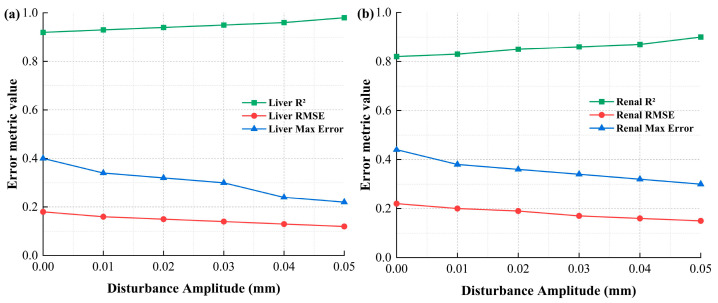
Effect of lateral disturbance amplitude on simulation accuracy for different porcine tissues: (**a**) liver and (**b**) renal tissues.

Figure 12 compares the simulation errors for both porcine liver and renal tissues under different disturbance amplitudes. As shown in Figure 12a,b, the simulation error metrics—RMSE, R^2^, and Max error—demonstrate distinct trends for the two kinds of soft tissues. Notably, as the disturbance amplitude increases, the simulation accuracy decreases, with porcine liver tissue exhibiting greater sensitivity to lateral disturbances than porcine renal tissue. These findings emphasize the need for improved disturbance modeling to enhance simulation robustness, particularly in replicating the dynamic conditions encountered during the puncture process. The results underscore the importance of considering such disturbances when refining the theoretical model to achieve more accurate predictions in real-world scenarios.

The above results also suggest that, while the current model provides a reasonable approximation of the puncture process, further optimization is required. Further efforts should focus on enhancing the material models to incorporate more detailed tissue characteristics, improving mesh resolution in the simulations, and considering additional variables such as friction, tissue heterogeneity, and external perturbations. These concerns will collectively improve the model’s accuracy and predictive capability.

The predictive accuracy of our proposed multi-component model shows a significant improvement compared to traditional static or single-component models. For instance, Jushiddi et al. [38] developed a multilayer model for liver puncture with an R^2^ of approximately 0.88–0.92; our model achieves higher fidelity by incorporating lateral perturbation effects that more closely mimic the micro-vibrations in robotic-assisted scenarios. Furthermore, while Trączyński et al. [31] highlighted the importance of viscoelasticity at varying speeds, our integration of the Maxwell model for renal tissue successfully captured the specific force-gradient fluctuations across anatomical layers that are often smoothed over in simplified hyperelastic models. Unlike the work of Abolhassani et al. [34], which primarily focused on axial friction, our results demonstrate that lateral disturbances, though small in amplitude, are essential for characterizing the high-frequency ‘force noise’ observed in experimental data, thereby providing a more robust foundation for real-time haptic feedback systems.

## 6. Conclusions

This study presents a dynamic force model for soft tissue puncture, integrating both experimental and simulation-based approaches. The proposed model successfully accounts for the complex interaction mechanisms between the needle and soft tissue, including cutting, stiffness, and frictional forces. Through systematic experiments performed on porcine liver and kidney or porcine renal tissues, the effect of insertion speed and angle on the force response was quantitatively characterized, and the simulation results were validated with experimental data.

Some conclusions of this study are presented below.

The findings indicate that both insertion speed and angle significantly affect the puncture forces. Higher insertion speeds lead to increased force magnitudes and steeper force–time curves, whereas lower speeds result in a more gradual force buildup. The impact of soft tissue structure was also apparent, with the renal’s distinct anatomical regions influencing the force behavior in different stages of the puncture process, which highlights the tissue-specific nature of mechanical response. The analyses also reveal that the current simulation model, while offering reasonable predictions, requires further refinement to better capture the nonlinear elasticity, tissue deformation, and other complex behaviors of soft tissues, especially under high-speed puncture conditions. In addition, the introduction of lateral disturbances in the simulation, representing micro-vibrations from robotic arms, provides a more realistic modeling framework for micro-manipulation scenarios. The results show that the simulation’s accuracy decreases with increasing disturbance amplitude, particularly for liver tissue. This emphasizes the need for improved disturbance modeling to enhance simulation robustness, especially in real-world puncture environments.

Finally, the established model and validation strategy can provide a valuable foundation for developing intelligent robot-assisted puncture systems and high-fidelity simulation-based training platforms. In the future work, authors will focus on optimizing the material models, improving simulation resolution, and refining the disturbance compensation mechanisms to enhance both the accuracy and applicability of the model in clinical practice and robot-assisted surgical applications.

## Figures and Tables

**Table 2:** Range of factors.

Factors	Range of Factor Values		
Puncture Speed	0.5 mm/s	1.5 mm/s	2.5 mm/s
Puncture Angle	15°	30°	45°

**Table 3:** Effects of different speeds and angles on the results of porcine liver and renal tissues puncture experiments: ANOVA and Tukey HSD test.

Regression Parameters	ANOVA and Tukey’s HSD Parameters				
	Meandiff	*p*-adj	Lower	Upper	Reject
a_1_	0.1968	0.0274	0.0584	0.3352	True
a_2_	0.3376	0.0001	0.1992	0.476	True
a_3_	0.1408	0.0251	0.0024	0.2792	True
a_4_	−0.0821	0.0154	0.1321	0.0331	True
a_5_	−0.207	0.0001	−0.3222	−0.0919	True
a_6_	−0.1249	0.0297	−0.2401	−0.0098	True
a_7_	0.135	0.0001	0.0588	0.2112	True
a_8_	0.2649	0.0001	0.1887	0.3411	True
a_9_	0.1299	0.0002	0.0537	0.2061	True
a_10_	−0.1568	0.0001	−0.218	−0.0957	True
a_11_	−0.2746	0.0002	−0.3357	−0.2134	True
a_12_	0.1177	0.0003	−0.1789	−0.0566	True

## Data Availability

Data is contained within the article, and detailed data plots for figures can be found in the article.

## Abbreviations

The following abbreviations are used in this manuscript:

FE	Finite Element
R^2^	Coefficient of Determination
RMSE	Root Mean Square Error
ANOVA	Analysis of Variance
HSD	Honestly Significant Difference
